# Acquired nasolacrimal duct obstruction: clinical and histological findings of 275 cases

**DOI:** 10.1186/s12886-021-02185-x

**Published:** 2022-01-05

**Authors:** Almantas Makselis, Donatas Petroska, Aiste Kadziauskiene, Ruta Jaruseviciene, Andrius Ruzgys, Andrius Cimbalas, Justinas Besusparis, Rimvydas S. Asoklis

**Affiliations:** 1 Centre of Eye Diseases, Vilnius University Hospital Santaros Klinikos, Santariskiu 2, LT-08661 Vilnius, Lithuania; 2 Center of Eye Diseases, Vilnius University, Santariskiu 2, LT-08661 Vilnius, Lithuania; 3 National Center of Pathology, affiliate of Vilnius University Hospital Santaros Klinikos, P. Baublio 5, LT-08406 Vilnius, Lithuania; 4 Faculty of Medicine, Vilnius University, M. K. Ciurlionio 21, LT-03101 Vilnius, Lithuania

**Keywords:** Dacryocystitis, Dacryocystorhinostomy, Lacrimal sac tumors, Lacrimal sac biopsy

## Abstract

**Background:**

Acquired nasolacrimal duct obstruction is a blockage of the lacrimal outflow system usually caused by local nonspecific inflammation of the lacrimal sac and the nasolacrimal duct. However, cases exist where the primary nasolacrimal system obstruction is caused by malignancies.

Our aim was to investigate lacrimal sac pathologies in patients with acquired nasolacrimal duct obstruction and compare their clinical manifestations.

**Methods:**

This retrospective clinical study included 275 patients with acquired nasolacrimal duct obstruction who underwent external dacryocystorhinostomy and lacrimal sac biopsy. Cases were classified into tumor or nonspecific pathology groups and subdivided according to the level of inflammation. Histological and clinical data were analyzed.

**Results:**

Three tumors (1.1%) (an adenoid cystic carcinoma, an eccrine spiradenoma and small B cell lymphoma) were diagnosed. Chronic nongranulomatous inflammation was the most common histological finding, corresponding to 194 cases (70.5%). The other 81 (29.5%) were subacute, acute forms of nongranulomatous inflammation, tumors and fibrosis cases. Epiphora with continuous purulent discharge was the most common clinical sign reported by 144 (52.4%) patients, and two (0.7%) patients had a palpable mass near the medial canthal tendon, which was identified as an eccrine spiradenoma and small B cell lymphoma. There was no significant difference in the clinical symptoms, duration or case history between the nonspecific pathology and tumor groups (*p* = 0.292).

**Conclusions:**

Chronic nongranulomatous inflammation of the lacrimal sac was the most common finding among acquired nasolacrimal duct obstruction cases. There were no associations between the histological findings and clinical presentation. The authors recommend a lacrimal sac biopsy only in cases when a tumor is clinically suspected.

## Background

Acquired nasolacrimal duct obstruction (ANDO) is a common disease of the lacrimal passages that is most frequently caused by local nonspecific inflammation of the lacrimal sac and the nasolacrimal duct, resulting in occlusive fibrosis [1, 2]. The clinical symptoms include chronic lacrimation that is aggravated by exposure to sun, wind, or cold.

Secondary causes of ANDO include lacrimal sac neoplasia, inflammatory diseases, specific infections, mechanical obstruction, and trauma [3]. Most frequently, tumors of the lacrimal sac are malignant and arise from squamous cells or the glandular epithelium [4]. Bloody discharge from a lacrimal duct and the presence of a palpable mass in the area of a lacrimal sac are suggestive of a malignant tumor. However, up to 40% of all nasolacrimal duct tumors may be undiagnosed and confused with primary ANDO and/or chronic dacryocystitis [5–7].

According to some authors, to ensure the timely diagnosis of tumors involving the lacrimal drainage system, a routine biopsy and histopathological examination of the lacrimal sac should be performed for all patients undergoing dacryocystorhinostomy (DCR) [4, 8–10]. However, other researchers recommend biopsies only in select cases in which there is clinical or intraoperative suspicion of a tumor because unsuspected tumors causing ANDO are relatively rare, with an incidence ranging from 0 to 7.1% [9–12].

## Methods

This retrospective interventional clinical study was carried out at the Center of Eye Diseases of Vilnius University Hospital Santaros Klinikos from January 1, 2010, to January 1, 2021. Histological and clinical data from the patients who underwent external DCR surgery due to ANDO were analyzed. Patients with previous nasolacrimal duct trauma were excluded. The study was conducted on the basis of the guidelines of the Declaration of Helsinki with the approval of the institutional bioethical committee. Informed consent was obtained from the participants.

The diagnosis of ANDO was confirmed by probing and irrigating the lacrimal drainage system. Each patient’s demographic and clinical data (presenting symptoms, duration of the symptoms), as well as disease history (episodes of acute dacryocystitis, previous nasolacrimal system surgeries), were recorded. In total, 275 external DCRs were performed by three surgeons (A. M., A. R., and J. A.) using an external approach with an osteotome or bone drill, followed by canalicular silicon intubation.

**Fig. 1:**
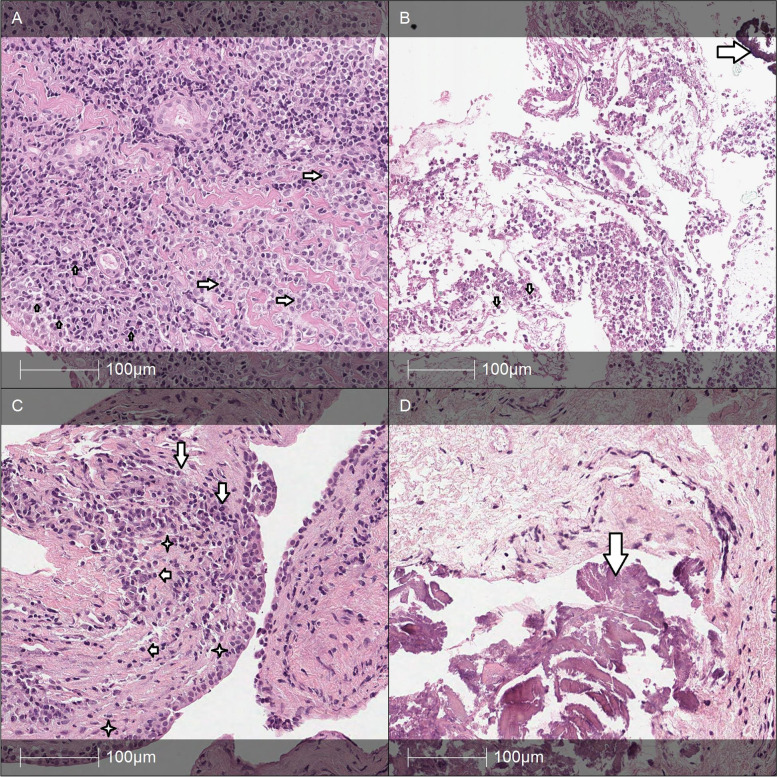
Hematoxylin/eosin (H/E) staining of the specimens. **A** Chronic inflammation: dense infiltration of plasmocytes (arrows) with some lymphocytes (short arrows in the lower right part) under a desquamated epithelium. H/E. **B** Acute inflammation: necrotic debris with some epithelium (in the center), granulocytes (below; short arrows), and calcification granules (arrow in the upper part). H/E. **C** Active chronic inflammation: mixed inflammation (plasmocytes – arrows, lymphocytes – short arrows, granulocytes – arrowheads) in the subepithelial stroma and some intraepithelial granulocytes (stars). H/E. **D** Fibrosis: fibrotic tissue with calcification (arrow) in the center without any inflammatory cells. H/E

Biopsy specimens were obtained from the medial-posterior wall of the lacrimal sac after full visualization of the internal sac structures, and they were preserved in a 10% formalin solution. Histological specimens were prepared from paraffin blocks and stained with hematoxylin/eosin. The specimens were also subjected to histochemical staining with Alcian blue and immunohistochemical staining with antibodies against the cytokeratins AE1/AE3 (Dako), CK5 (XM26, Novocastra), p63 (4A4, Dako), and Ber-Ep4 (Dako), as well as CD117 (c-kit p145, Dako) and CD43 (DF-T1, Dako). All the tumors found in this study were stained for Ki67 (MIB-1, Dako) to determine the proliferative index. All histopathological findings were allocated to either a nonspecific pathology or a tumor group. The specimens with nonspecific pathology were further divided into groups of chronic, acute, or subacute inflammation or fibrosis on the basis of the presence of elements of an active inflammatory process (Table 1, Fig. 1).

**Table 1:** The histopathological categories of the nonspecific pathology group

Group 1	Chronic inflammation	Lacrimal sac wall infiltrated by lymphocytes and plasmocytes with no more than 1% of segmented leukocytes in a fibrous stroma (Fig. 1A).
Group 2	Acute inflammation	Infiltration consists of >90% segmented leukocytes in edematous nonfibrotic tissue or in fibrous tissue (Fig. 1B).
Group 3	Subacute inflammation	Infiltration of tissue by mixed segmented leukocytes (<90% of inflammatory cells), plasmocytes and lymphocytes in a fibrous tissue (Fig. 1C).
Group 4	Fibrosis	Fibrous tissue without inflammatory infiltration; calcification (Fig. 1D).

Data were analyzed using R commander software (R program software version 3.1.2, CRAN Project). Fisher’s exact test was used to compare clinical data among various histological findings. *p* < 0.05 was considered statistically significant.

## Results

A total of 258 patients (275 cases of DCR) were enrolled in the study, and their clinical and pathological findings were analyzed. The study participants included 189 (73,3%) females and 69 (26,7%) males. The mean age of the patients was 69.7 years (SD 13.1, range 25–98).

Histopathological analysis revealed three (1.1%) specific pathologies: an adenoid cystic carcinoma, an eccrine spiradenoma and small B cell lymphoma. A total of 218 (79.2%) cases showed nonspecific inflammation, and 54 (19.6%) demonstrated noninflammatory fibrotic changes. Chronic nongranulomatous inflammation was the most common finding and was detected in 194 (70.5%) of the specimens (Table 2).

**Table 2:** The results of histological analysis

Nonspecific pathology Cases (%)				Specific pathology Cases (%)
Nongranulomatous inflammation			Fibrosis	Tumor
218 (79.2)			54 (19.6)	3 (1.1)
Chronic	Acute	Subacute		
194 (70.5)	14 (5.1)	10 (3.6)		

Chronic lacrimation was observed in 213 (77.5%) of the patients, with a median duration of 24 (median; interquartile range (IQR) 48.0) months. Purulent discharge was documented in 169 (61,5%) of the patients and lasted for 12 (median; IQR 20.4) months. A total of 144 (52.4%) of the patients complained of both epiphora and continuous purulent discharge, while 69 (25.1%) reported only epiphora, and 25 (9.1%) reported only purulent discharge. The overall median duration of the symptoms was 12 (median; IQR 21) months. In 2 patients, a palpable mass in the region of the lacrimal sac was observed, and after histological analysis, the mass appeared to be an eccrine spiradenoma and small B cell lymphoma.

Three patients received repeated external DCR for the same eye, and 10 received DCR for the other eye. An external incision was made for 3 patients in the same eye and for 1 patient in the contralateral eye before the study due to acute dacryocystitis or lacrimal sac abscesses.

No significant difference was found in the clinical symptoms between the patients with nonspecific histological findings and those with tumors (*p* = 0.292) (Table 3).

**Table 3:** The clinical symptoms according to histological findings

	Lacrimation	Purulent discharge	Previous ipsilateral DCR	Previous contralateral DCR
Patients (%)				
Chronic inflammation	153/194 (78.9)	120/194 (61.9)	5/194 (2.6)	15/194 (7.7)
Acute inflammation	13/14 (92.9)	11/14 (78.6)	0/14 (0)	0/14 (0)
Subacute inflammation	8/10 (80.0)	7/10 (70.0)	0/10 (0)	1/10 (10.0)
Fibrosis	37/54 (68.5)	29/54 (53.7)	3/54 (5.56)	4/54 (7.4)
Tumor	2/3 (66.7)	2/3 (66.7)	0/3 (0)	0/3 (0)

% - Percentage of all patients in the nonspecific pathology group

The patients did not differ significantly in the duration of the symptoms according to their histological findings (*p* = 0.331). The median duration of symptoms was longer in the tumor group. Lacrimation and purulent discharge in the tumor group lasted 30 (IQR 12) and 18 (IQR 12) months, respectively. In all patients with tumors, there was no history of preceding acute dacryocystitis or external DCR. However, the differences in the history of lacrimal pathology between the tumor group and the nonspecific pathology group were not statistically significant.

The cases of three patients with tumors are described below.

### Case 1

**Fig. 2:**
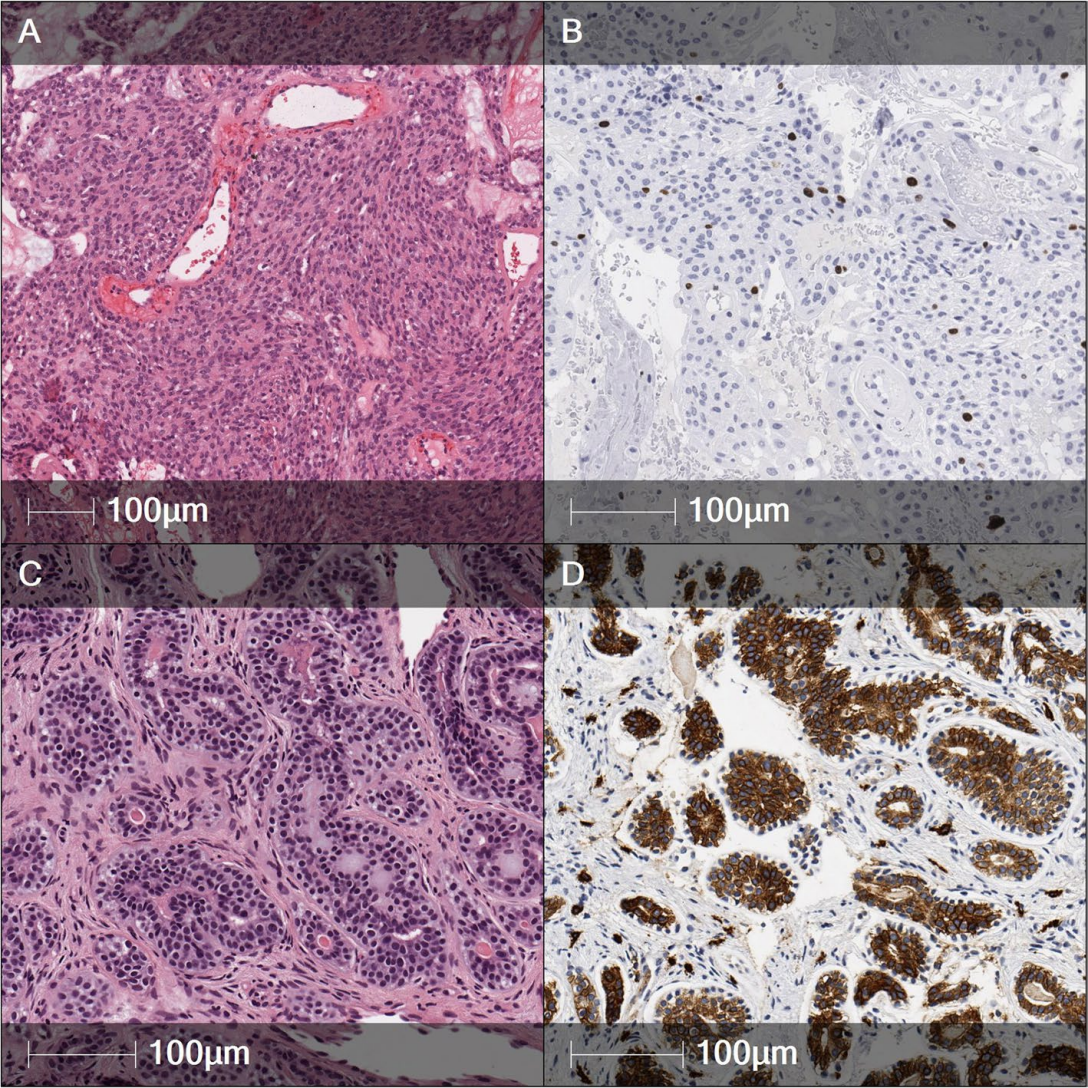
Tumor histological findings. **A**. Eccrine spiradenoma. Magnified image of hematoxylin/eosin (H/E)-stained tissue. **B**. The epithelial cell Ki67 proliferative index of the eccrine spiradenoma was 4%. **C**. Adenoid cystic carcinoma. Magnified image of H/E-stained tissue with secretion in the ductal structure lumens. **D**. Adenoid cystic carcinoma cells were positive for CD117

An 84-year-old woman presented with tearing and purulent discharge associated with a solid palpable mass below the medial canthal tendon lasting for two years. The neoplastic process was suspected. Magnetic resonance imaging (MRI) revealed a 16 x 18 x 16 mm mass in the region of the upper nasolacrimal duct with intensive nonhomogeneous contrast enhancement and no signs of local invasion. A biopsy of the lacrimal sac confirmed the diagnosis of an eccrine spiradenoma. Histological analysis showed vascularized masses of compact solid and trabecular microcystic structures composed of cuboidal and cylindrical cells with clear cytoplasm and polymorphic nuclei, with a Ki67 proliferative index of 4% (Fig. 2A, B) and negative staining for S100 and CD43. The patient was monitored by hematologists constantly, and the disease was stable. There are no data about any interventions or surgeries later after diagnosis.

### Case 2

A 65-year-old woman complained of epiphora lasting for 15 years and recent symptoms of itching and swelling of the eyelids. The patient had a history of bilateral maxillary sinus surgery due to sinusitis 20 years earlier. Histological analysis revealed an adenoid cystic carcinoma. The tumor was verified by histochemical and immunohistochemical methods, demonstrating that the lumens of the glands and cribriform structures composed of epithelial cells had Alcian blue-positive mucins and that the epithelial cells were positive for CD117 (90%) and CD43 (10%) and the cytokeratins AE1/AE3, CK5, p63, and Ber-Ep4 (Fig. 2C, D). The Ki67 proliferative index was 30%, and the tumor was negative for synaptophysin.

### Case 3

**Fig. 3:**
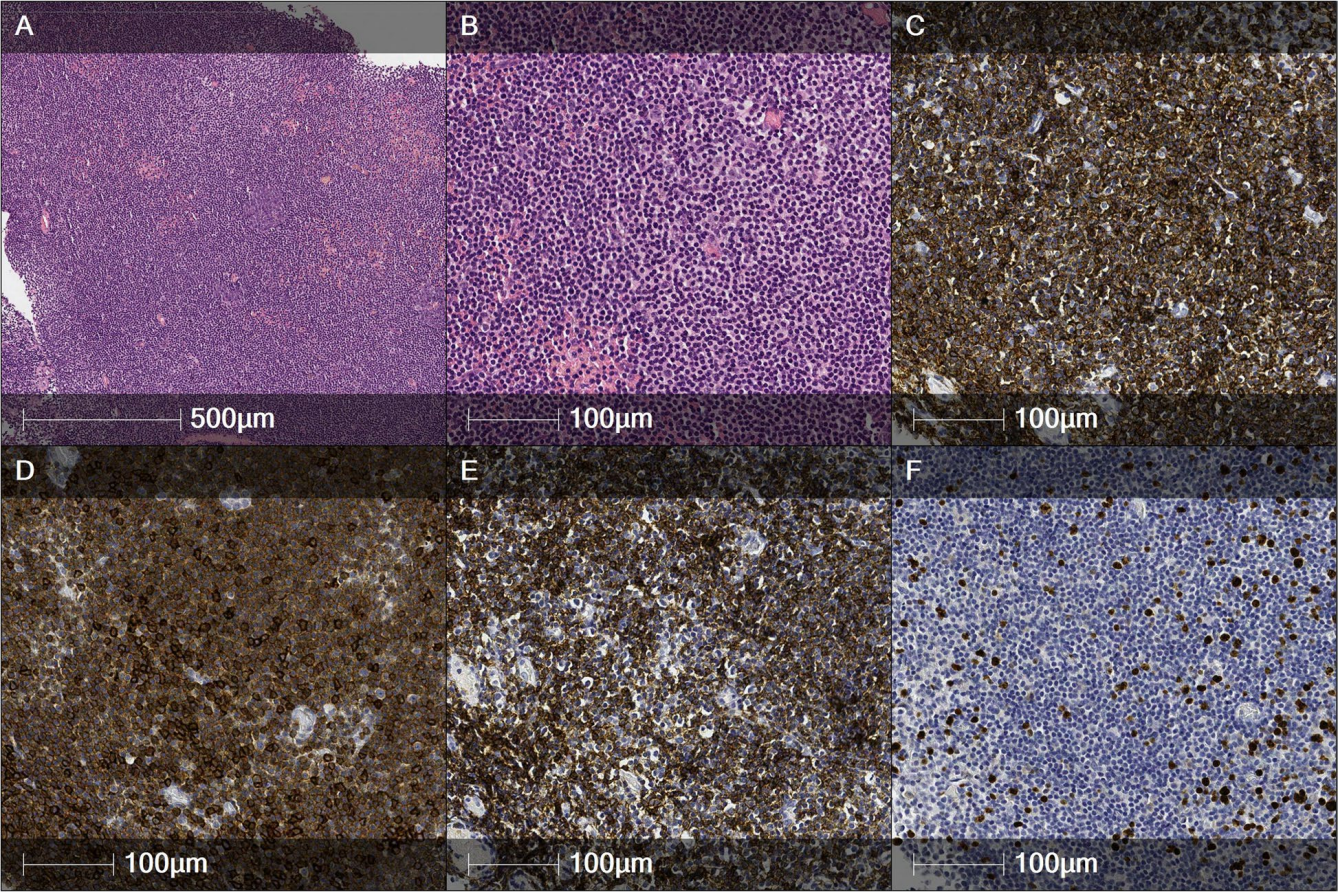
Chronic lymphocytic leukemia/small lymphocytic lymphoma. **A-B** Tear sac tissue section illustrating a diffuse and focally nodular pattern of lymphocytic infiltration composed of small and round lymphocytes, with distinct clumped chromatin. (H/E). Immunophenotype: CD20+ (**C**), CD5+ (**D**), CD23+ (**E**), Ki67 proliferative activity <15% (**F**)

An 82-year-old woman complained of epiphora lasting for 2 years and purulent discharge lasting for 1 year. Half a year ago, she noticed a solid mass in the region of the lacrimal sac. During the observation, a solid mass of 10 x 6 mm, with fluctuation, was observed. Histological analysis revealed small B cell lymphoma with the immunophenotype CD20/CD23/CD5+ and Cyclin D1/CD10/Bcl6/(−) and 15% Ki67 proliferative activity 15%. In addition, the analysis showed that the lymphoma had spread to tear sac tissues (Fig. 3).

## Discussion

The major histologically identified cause of ANDO is nonspecific chronic inflammation resulting in a blockage of the lacrimal outflow system. In our study, ANDO was associated with nonspecific histological changes in 79.2% of the cases and most often showed chronic nongranulomatous inflammation. These findings are consistent with those reported in other series. Some studies report that no specific histological features were found in 98% of ANDO cases [4, 13]. In a study by other authors, chronic inflammation was diagnosed in 95% of specimens, and fibrosis was detected in 3.8% [10]. Other authors reported nonspecific pathology in 96.49% of cases [10].

Previous studies have demonstrated that secondary causes, including primary or secondary tumors, tumor-like lesions, inflammatory diseases, and mechanical obstruction of the lacrimal drainage system, represent the etiology of ANDO in 0–14.3% of cases [8, 9, 11, 12, 14, 15]. In our study, three specific histological findings associated with ANDO were identified after histopathological analysis of the lacrimal sac wall. They included three neoplastic lesions - an adenoid cystic carcinoma, an eccrine spiradenoma and one case of small B cell lymphoma. The prevalence of ANDO caused by neoplastic lesions was 1.1%, which is similar to the prevalence reported in other studies, although it is lower than the 8.2% of the cases of ANDO reported by other authors [4, 9, 10]. One possible explanation for the difference is that their specimens may have been selected using laboratory findings but not surgical records [9]. Eccrine spiradenomas are rare benign sweat gland tumors, and few cases involving the eyelids have been described [16, 17]. This type of tumor is specific for skin adnexa; therefore, it could be interpreted as a skin tumor with ANDO. The patient with a spiradenoma in our study did not complain of pain, which is common for this type of tumor [17]. A lacrimal sac adenoid cystic carcinoma is a rare malignant tumor that can be lethal [18]. Lacrimal sac lymphomas are rare and malignant tumors that are often left misdiagnosed. They present with symptoms of dacryocystitis and epiphora. There is a report that describes 3 cases of lacrimal sac leukemia/small-cell lymphocytic lymphoma [19]. Some authors found only 3 cases of adenocarcinoma among 74 malignant lacrimal sac tumors, and others reported 4 adenocarcinomas among 115 lacrimal sac neoplasms [5, 6]. According to previous studies, positivity for CD117 and CD43 is a sensitive and relatively specific marker of adenoid cystic carcinomas, as demonstrated in our study [20, 21].

ANDO due to neoplastic causes is reported to be relatively rare; however, overlooking this potentially lethal etiology can delay diagnosis until an advanced stage [6, 7, 22]. Our study demonstrated that the common clinical symptoms of ANDO, such as epiphora and purulence, did not allow differentiation between the neoplastic lesions and nonspecific etiology; however, a specific sign such as a solid palpable mass led to the suspicion of the tumor in the spiradenoma and small B cell lymphoma cases. Most of the primary tumors of the lacrimal sac are malignant, with mortality as high as 37.5%; thus, prompt diagnosis is important for effective treatment [6, 10]. In another study, an analysis of 82 cases of dacryocystic tumors showed that the primary diagnosis of dacryocystitis was established in 5% of patients; in 55% of patients, diagnosis was confirmed only when the tumor had already invaded the adjacent tissues, and in 18% of patients, the tumor was diagnosed when distant metastases were already present [6]. Another study examined 22 clinical cases of lacrimal sac tumors and found that in 27% of cases, the primary diagnosis was incorrect, and the patients were previously treated for dacryocystitis [7].

Imaging tools are essential in the diagnostic differentiation of nasolacrimal drainage system tumors from chronic inflammatory conditions because they can mimic neoplasms [23]. Computed tomography (CT) and MRI can be used for diagnostic and screening purposes. CT is more sensitive for the detection of bone destruction than MRI, but MRI has a high soft-tissue resolution. Both CT and MRI are suitable for determining tumor recurrence during patient follow-up.

Studies show that even without surgical removal of dacryocystic tumors (squamous cell carcinomas), patients might have achieved excellent long-term clinical outcomes, including 5-year overall survival, progression-free survival and locoregional control, with radiation therapy alone [24]. Considering the low frequency of cases with this specific etiology, many researchers advocate the biopsy of the lacrimal sac wall only when the patient’s medical history and clinical or intraoperative suspicion are present [4, 8–10]. However, this approach can potentially delay the diagnosis. In a Stage 1 tumor described by some authors, no palpable masses of the lacrimal sac were found, and no symptoms of ANDO were observed [6]. However, according to other authors, only 0.5% of 1294 cases were unsuspected when the specific pathologies of ANDO were diagnosed [14].

In our study, there was no significant difference in the disease history, clinical symptoms, or symptom duration between the patients with ANDO with specific and nonspecific pathology or among the patients with nonspecific inflammation according to histological findings, suggesting that the clinical context does not improve the identification of the cause of ANDO, and a biopsy is required. However, in cases of eccrine spiradenoma and small B cell lymphoma, the palpation of a hard, subcutaneous mass and the intraoperative appearance of the tissues of the lacrimal sac led us to suspect neoplastic disease. We did not observe a bloody discharge in any of the patients, as previously described [25, 26]. No suspicious clinical symptoms or intraoperative observations were noted in the patients with an adenocarcinoma of the lacrimal sac. On the basis of the finding of that only one case of preoperatively unsuspected malignant neoplasia was diagnosed histologically out of 275 specimens, the authors of this study recommend selective biopsy of the lacrimal sac when there is clinical or intraoperative suspicion of a tumor.

The first limitation of the study is the number of cases. A larger sample size could make it possible to more accurately evaluate the frequencies of the secondary changes in ANDO and its associations with the clinical appearance. An insufficient sample size and the fact that biopsy specimens were taken only from the medial-posterior wall of the lacrimal sac could be the reason why we did not find any specific inflammatory changes, such as sarcoidosis or Wegener granulomatosis, in our patients, as described in other studies [9, 10, 14]. Additionally, a larger sample size may have revealed a difference in the symptom duration between the tumor group and the nonspecific pathology group. The second limitation would be that data on lacrimal system irrigation would add value to this study by improving the preoperative evaluation of the patients.

The authors concluded that chronic nongranulomatous inflammation of the lacrimal sac wall was the most frequent histological finding in cases associated with ANDO; however, three cases of tumors were diagnosed, and two of them were potentially lethal. The complaints of the patients were nonspecific, and no associations among the histological findings, clinical presentation, and history of the disease were observed. Because the rate of malignant neoplasia was low, the authors support that a lacrimal sac biopsy is indicated only in clinically or intraoperatively suspected cases of ANDO.

## Data Availability

The datasets analyzed during the current study are available from the corresponding author on reasonable request.

## Abbreviations

ANDO: Acquired nasolacrimal duct obstruction
DCR: Dacryocystorhinostomy
IQR: Interquartile range
MRI: Magnetic resonance imaging
CT: Computed tomography
